# Experimental and numerical investigations on the failure processes and mechanisms of composite coal–rock specimens

**DOI:** 10.1038/s41598-020-70411-5

**Published:** 2020-08-07

**Authors:** Fuqiang Gao, Hongpu Kang, Lei Yang

**Affiliations:** 1 State Key Laboratory of Coal Mining and Clean Utilization (China Coal Research Institute), Beijing, China; 2 CCTEG Coal Mining Research Institute, Beijing, China

**Keywords:** Natural hazards, Solid Earth sciences

## Abstract

Brittle failure is a fundamental failure pattern in many different materials, from small nanoscale materials with single crystals to the large earth crust. Many efforts have been dedicated to understanding the brittle failure mechanisms of individual brittle and semi-brittle materials. Limited studies have been conducted on the brittle failure of composite materials with interaction and energy feedback between different materials. Here we investigated the brittle failure pattern of coal–rock composite materials under uniaxial compression by laboratory tests and numerical simulations. We used a high-speed camera to capture the failure of coal–rock specimens. For all three tested coal–rock combined specimens, the rock failed with a splitting pattern that resulted from a single tensile fracture that developed sub-parallel to the loading direction. We regarded this brittle failure as a sliding-induced tensile fracture from frictional drag that was caused by unequal lateral deformation of the rock and coal under identical axial loading. The tensile crack propagated stably at ~ 0.05 times the Rayleigh wave speed *c*_*R*_. We observed an unstable failure pattern of the coal samples that was characterized by the ejection of small pieces from the coal specimen surface. This behavior is attributed to the strain energy that is stored in the rock specimen, which releases when the coal fails. The excessive strain energy transitions into dynamic energy during coal failure. Our findings provide insight into the brittle failure mechanisms of composite materials and have significant implications at scales relevant to seismicity, engineering applications and geohazards.

## Introduction

Brittle failure is a fundamental failure pattern in many materials, from small nanoscale materials with single crystals^1–3^ to the large earth crust^4^. An understanding of the mechanisms of brittle failure and brittle–ductile transition is significant for science and engineering. Silicon is a typical single-crystal material that experiences brittle failure at the nanoscale. The prevention of brittle failure of silicon is of significant importance when the nanostructures are machined and fabricated for application in integrated circuits, solar cells and energy storage systems^5^. Investigations into the failure mechanisms with nanoscale deformation and high loading rates require sophisticated approaches for single-grain grinding/scratching^6–11^. Crustal rocks may contain several microstructures with a size that is approximately equal to the grain size. These microstructures promote brittle failure of rock materials when subjected to compressive loading. Brittle failure begins when wing cracks (i.e., secondary cracks) are created at the tips of microcracks (i.e., first cracks)^12^. The energy transition is essential at all scales of brittle failure. A brittle crack can propagate only if the input energy is greater than the fracture energy^13^. More energy is needed when plastic deformation occurs ahead of the crack tips^14–16^. The problem becomes more complicated for the brittle failure of multiple materials where energy feedback between different materials is involved in failure^17^. Such a typical problem includes strainbursts, which are violent excavation-induced events that involve the sudden failure and ejection of rocks from the surface of an underground excavation^18,19^. The dynamic energy of ejected rocks tends to originate from the energy that is stored in the zone where the bursts occur, and also from the surrounding rocks^20–22^. The violent destructive nature results because the inflow of external energy exceeds the energy absorption during destruction, and the excess energy is converted to the kinetic energy of flying fragments^23^. This energy-absorbing-transition concept can be illustrated by using the local mine stiffness (LMS) theory that was first proposed by Salamon^24^. LMS is defined as an equivalent stiffness of the roof, coal, and floor strata in different areas of the mine at different stages of mining^24^. If the LMS is too “soft” (i.e., lower than the post-peak pillar stiffness), the strain energy that is stored in the roof and floor is released and transitions into the coal where it causes burst failure. Chen et al.^25^ proposed a double rock sample model to represent the soft loading conditions where one rock was used to simulate the failed zone of a rock and the other was used to simulate the surrounding rocks. Since then, many researchers have used double rock samples to investigate brittle failure^26–31^. Common conclusions from there studies include (1) the mechanical properties and deformation failure characteristics of coal–rock specimens are governed mainly by the coal, which is weaker than the rock; (2) coal–rock specimens exhibit greater bursting liability indices; and (3) the rock sample tends to fail in a tensile splitting pattern, which is attributed to the energy released from coal failure.

This research has provided an understanding of the mechanical response characteristics and brittle failure modes of coal–rock specimens under different loading conditions. Limited efforts have been dedicated to the failure of composite coal–rock specimens, particularly with unstable brittle failure. In this study, we used a high-speed camera to film coal–rock specimens that were subjected to uniaxial compression. We performed numerical simulations to demonstrate the failure pattern of coal–rock specimens that were studied in the laboratory and investigated the energy-absorbing-transition process that is associated with the unstable failure of coal in the coal–rock specimens.

## Experiments

The sandstone and coal samples that were used in this study were from Kuangou coal mine in the Xinjiang Uygur Autonomous Region, China. Sandstone and coal blocks were from the working face of a longwall entry at the mine and were transported to the laboratory. Sandstone and coal specimens were cored with bedding planes perpendicular to the long axis. The ends of the specimen were surfaced using a rotating grinding device. We prepared two sandstone and coal specimen types with a standard size of a 50-mm diameter and 100-mm length and a short size of 50-mm diameter and 50-mm length. Three sandstone specimens and four coal specimens with a standard size were prepared. These specimens were used in uniaxial compression tests to obtain the mechanical properties according to the methods suggested by International Society of Rock Mechanics (ISRM)^32^. The test results are listed in Table 1. The mean values of the uniaxial compressive strength (UCS), Young’s modulus E, and Poisson’s ratio were 33.23 MPa, 2.51 GPa and 0.26, respectively, for the coal, and 65.53 MPa, 12.54 GPa and 0.27, for the sandstone, respectively.

**Table 1:** Mechanical properties of the coal and sandstone used in this study.

Specimen	No	UCS/MPa	*E*/GPa	υ
Sandstone	R-1	57.68	11.35	0.24
	R-2	67.26	12.92	0.27
	R-3	71.65	13.36	0.31
	Mean	65.53	12.54	0.27
Coal	C-1	30.61	2.43	0.23
	C-2	27.37	2.51	0.23
	C-3	38.79	2.41	0.30
	C-4	36.13	2.68	0.27
	Mean	33.23	2.51	0.26

UCS is uniaxial compressive strength,* E* and υ are the Young's modulus and Poisson’s ratio, respectively.

**Figure 1:**
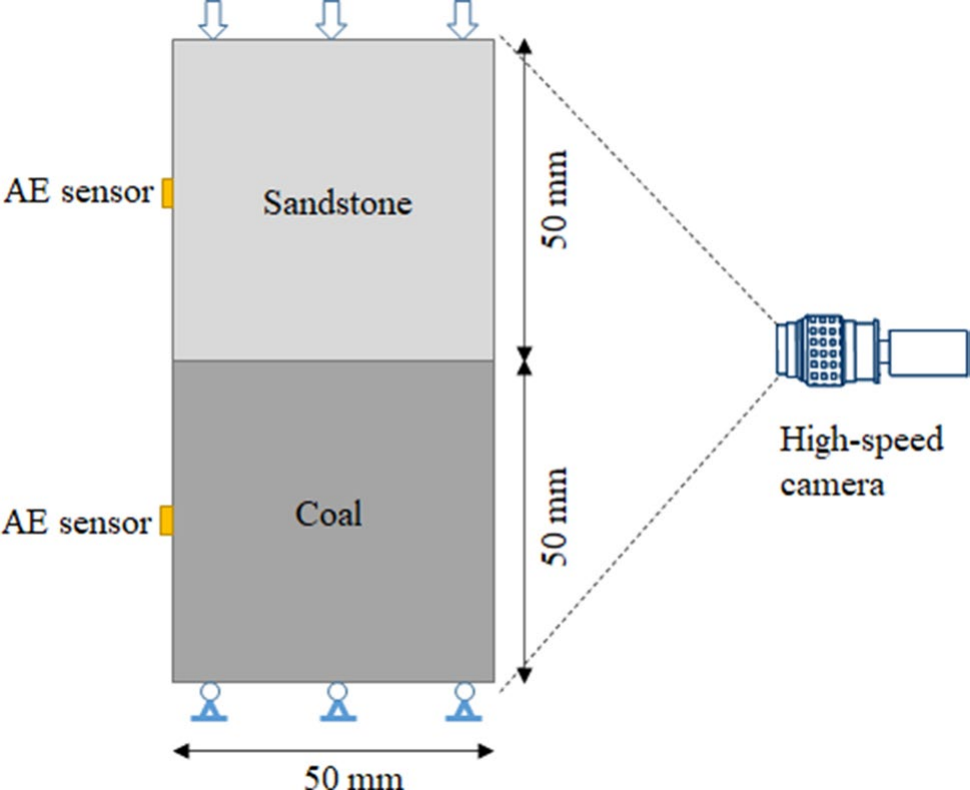
Configuration of the unconfined compression test on coal–rock specimens.

We prepared coal–rock specimens by combining a short sandstone sample and a short coal sample. No treatment was undertaken on the coal–sandstone specimen interface, which represents a dry and clean coal–rock joint. The coal–rock specimens were 100 mm long. Three coal–rock specimens were prepared for uniaxial compression tests, see Fig. 1. Two cylindrical plates that incorporated a spherical seat were placed between the specimen and a uniaxial compression loading frame. The specimen was loaded under uniaxial compression with a sufficiently low loading rate of 0.001 mm/s. A high-speed camera i-SPEED 726 (iX Cameras) was placed in front of the specimen to record the failure of the coal–rock specimens with a frequency of 40,000 fps. One acoustic-emission (AE) sensor was placed on each of the coal and sandstone samples to capture AE signals during loading. The sensors were placed at the rear of the coal–rock specimens so that they did not hinder the high-speed camera from capturing the specimen failure, see Fig. 1. Because of the limitations of the space position, it was impossible to arrange enough (at least 4) sensors to localize the AE events. Moreover, because of the different speeds of the sound waves that propagated in the coal and rock, it was difficult to locate the AE events precisely by using traditional methods.

## Results and discussion

**Figure 2:**
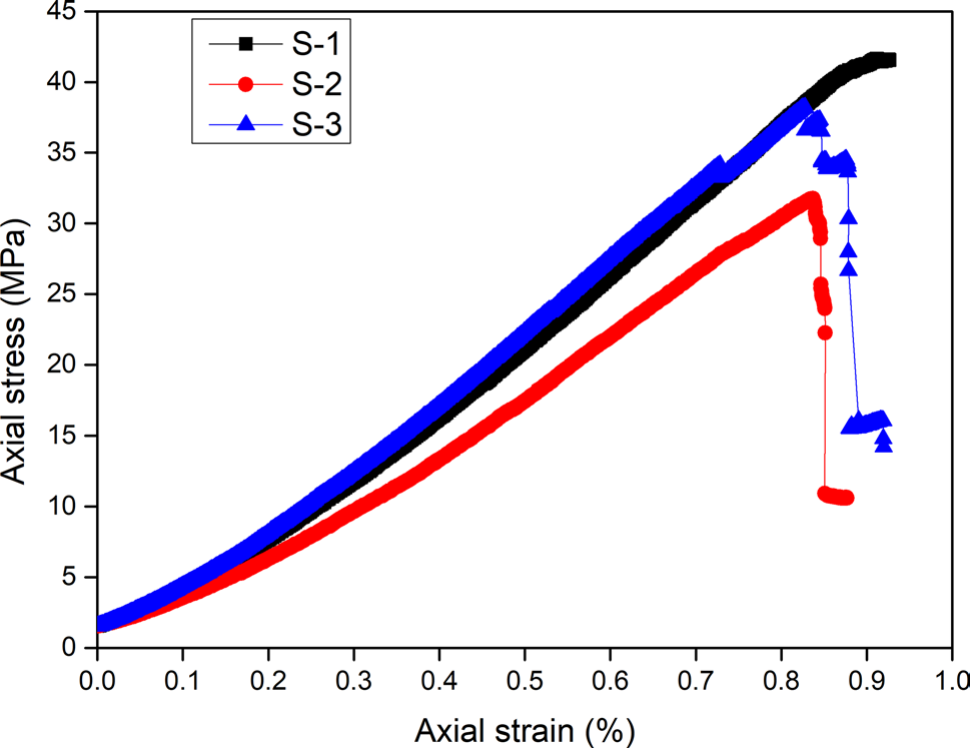
Axial stress–strain curves from unconfined compression tests on coal–rock specimens.

Figure 2 shows the axial stress vs. axial strain curves from the uniaxial compression tests on the coal–rock specimens. All three curves showed small deviations from linearity before the peak loads, which indicates a limited amount of plastic relaxation^3^. All three specimens exhibited brittle post-peak failure behavior (Specimen S-1 failed too rapidly and the post-peak curve was not obtained).

**Figure 3:**
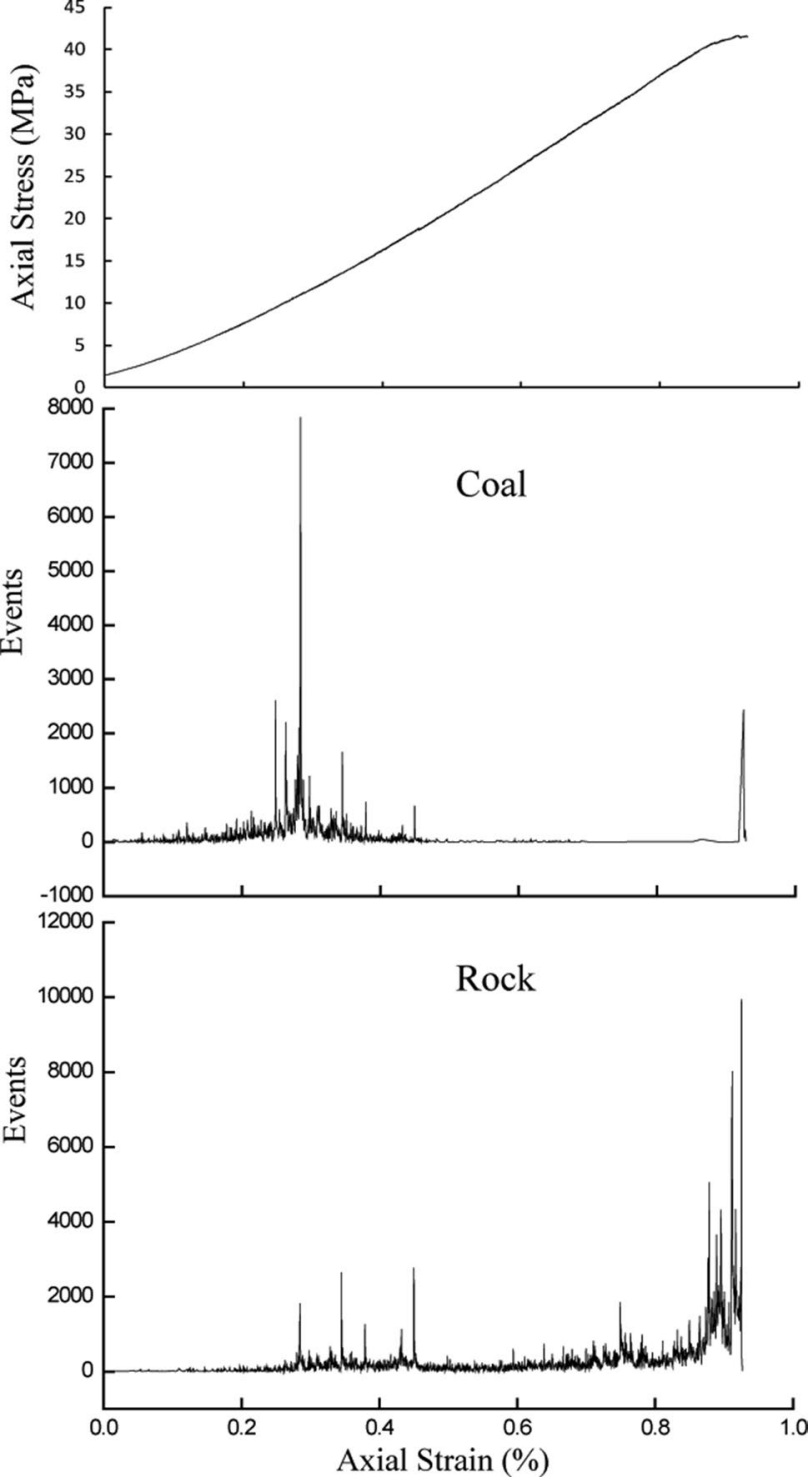
Changes in acoustic events in the coal–rock specimen S-1 under uniaxial compression. Events were monitored by two sensors on the coal and rock sample surface, respectively.

Figure 3 shows the changes in monitored AE events in coal–rock specimen S-1. Each sensor monitored the AE events in the entire coal–rock specimen, but because of the existence of the coal–rock interface and the different physical and mechanical properties of the coal and the sandstone, the AE sensor that was fixed on the coal sample surface was more likely to monitor the AE events that occurred in the coal than those that occurred in the sandstone. The sensor that was fixed on the sandstone sample surface was more likely to monitor the AE events that occurred in the sandstone. During the early loading stage (i.e., the axial strain was less than 0.2%), many pre-existing cracks in the coal sample were closed because of the loading, which generated AE events. These events were weak and were not monitored by the sensor that was fixed on the sandstone sample surface. The number of AE events increased significantly before the peak load.

The obtained UCS values for the three coal–rock specimens were 41.7, 31.8 and 38.2 MPa, respectively. The obtained Young’s modulus was 4.9, 4.4 and 4.9 GPa, respectively. The compressive strength of the coal–rock specimens tended to be slightly greater than the pure coal and significantly less than the pure sandstone, except for specimen S-2, where the strength of the coal–rock specimen was slightly less than that of the pure coal. This outcome likely resulted because of pre-existing cracks within the coal sample. Failure occurred mainly within the coal samples. The compressive strength of the coal–rock specimens was dominated by the coal sample, which was weaker than the rock. Two explanations for the coal–rock specimen being stronger than the pure coal specimen include that the height-to-diameter ratio of the coal sample in the coal–rock specimen was 1:1, which is less than the 2:1 of the pure coal specimen. Therefore, the end effect for the coal sample of the coal–rock specimen exceeded that of the pure coal specimen. The other explanation is that the possibility of the existence of critical cracks within the coal sample of the coal–rock specimens was lower than that of the pure coal specimen.

The mean value of Young’s modulus of the three coal–rock specimens was 4.7 GPa, which is significantly greater than the 2.5 GPa of the pure coal. The contribution of the sandstone sample to the deformability of the coal–rock specimen exceeded that of the strength.

### Failure mechanisms of rock

**Figure 4:**
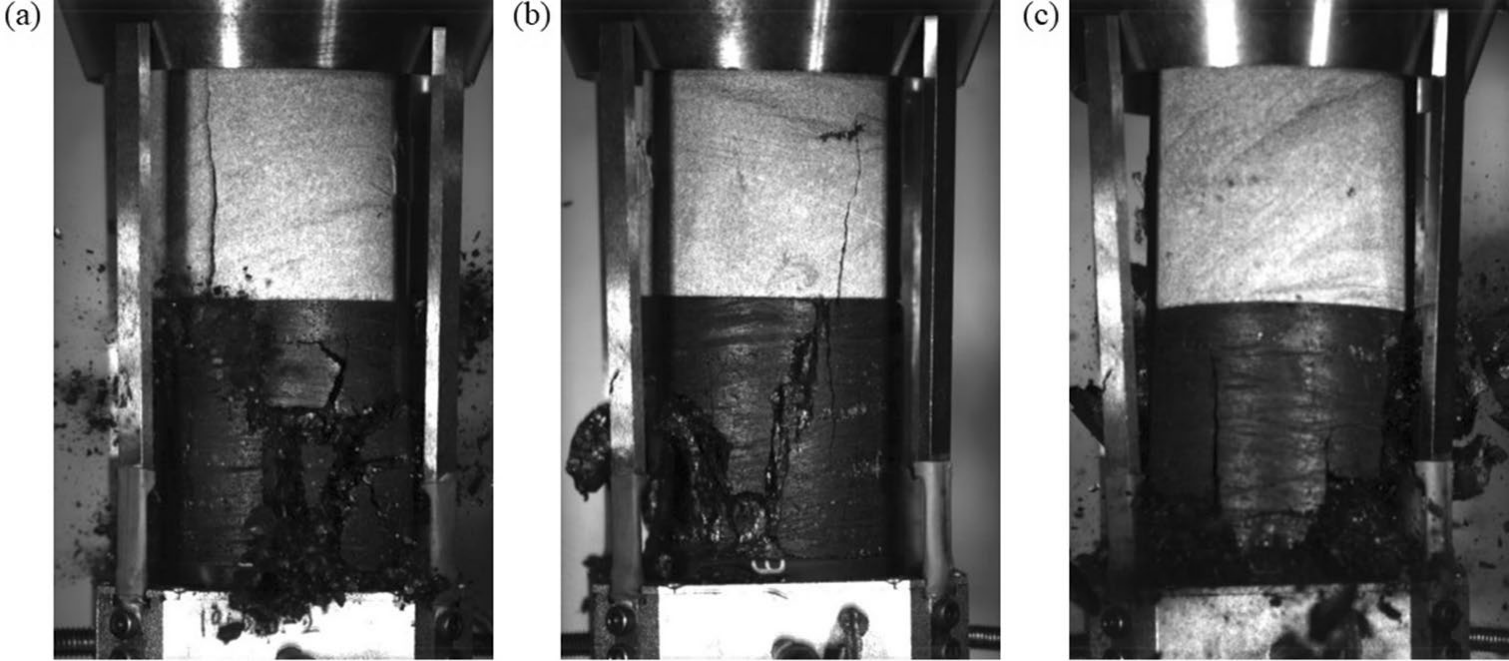
Failure patterns of coal–rock specimens subjected to unconfined compression.

Figure 4 shows the final failure patterns of the three coal–rock specimens that were subjected to uniaxial compression. For all three tested specimens, the sandstone samples failed in the same pattern that was characterized as a single tensile fracture that cut through the entire sample height.

**Figure 5:**
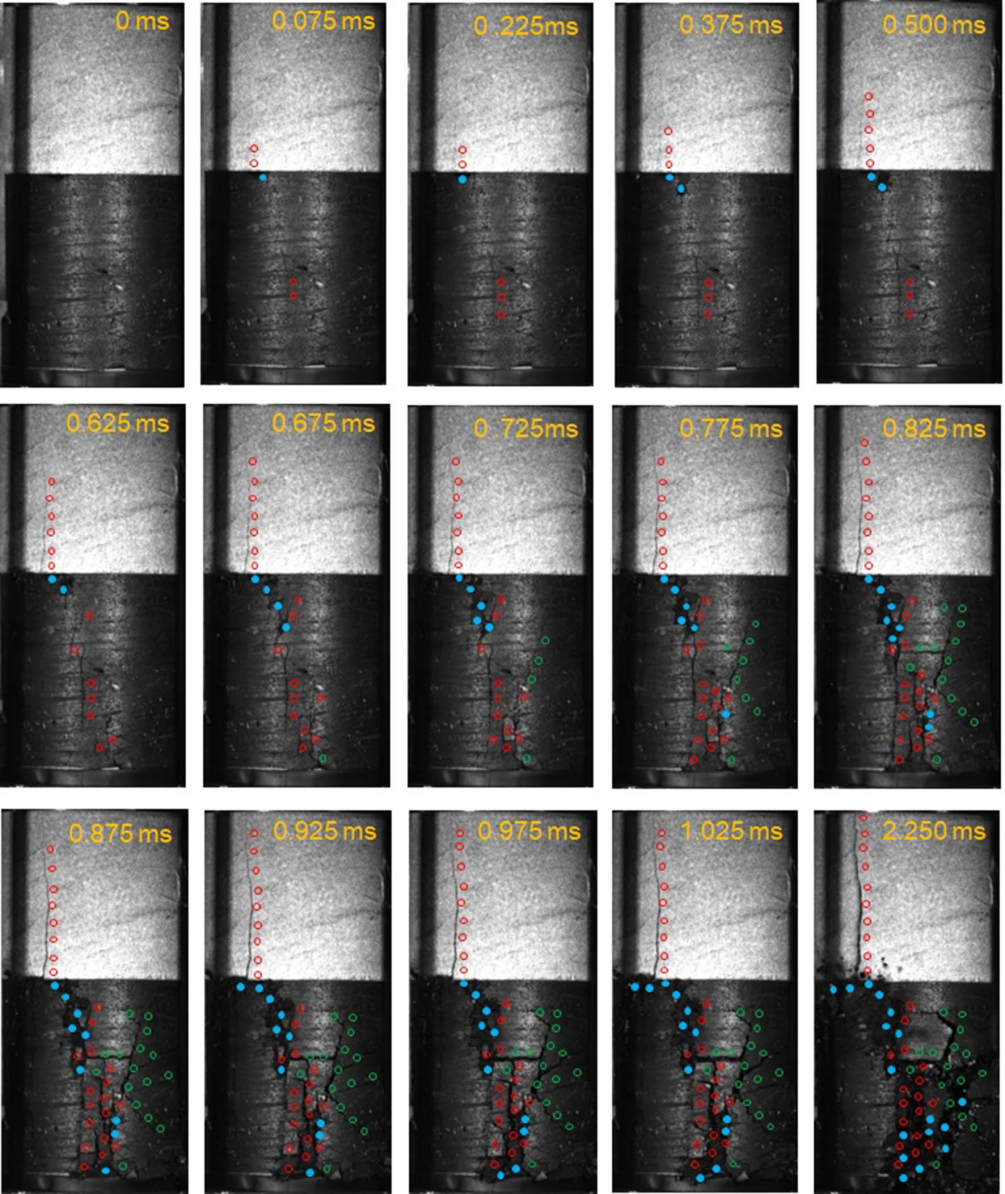
Failure of the coal–rock specimen S-1 captured by a high-speed camera. Red circles indicate tensile cracks, green circles indicate shear cracks, blue dots indicate the area where coal ejection occurs.

If we consider that, for each specimen, the peak stress was significantly less than the uniaxial compressive strength of the sandstone, the failure mechanism of the sandstone samples in the coal–rock specimens must differ from the standard sandstone specimens that were subjected to uniaxial compression. This extensile fracture pattern of the rock samples in the coal–rock specimens has been observed previously^28–30,33,34^ where it was found that the extensile fracturing of the sandstone sample was caused by energy released from coal sample failure or the extension of cracks within the coal. In this study, coal–rock specimen failure was captured by a high-speed camera. Figure 5 shows the snapshots of the failure of specimen S-2. The tensile fracture that initiated at the coal–rock interface propagated upwards at ~ 50 m/s, which is ~ 0.05 times the Rayleigh wave speed *c*_*R*_. This speed was significantly less than the limiting crack velocity that equals *c*_*R*_, which was predicted by classical continuum theory^35,36^. It was also less than the experimentally-observed maximum crack speeds of dynamic cracks that equal 0.3–0.4*c*_*R*_^14,37,38^. The crack speed of the tensile fracture that was generated in the sandstone sample of specimen S-1 was in the same range. This result suggests that the tensile fracture that was generated in the sandstone samples was stable. Figure 5 shows that the propagation of tensile fractures within the sandstone occurred before the coal failure. Thus, the tensile fracture was not caused by the energy that was released from the coal sample.

The tensile fracture was located at the core and cut through the height of the sandstone sample, which is distinct from spalling failure at the near-surface of the sandstone. The Young’s modulus of the sandstone was significantly greater than the coal (12.5 GPa vs. 2.5 GPa). However, the Poisson’s ratios of the sandstone and the coal were similar (0.27 vs. 0.26). The lateral strain of the coal sample ε_coal_ would exceed that of the sandstone sample under the same axial load (i.e., ε_coal_≈5ε_rock_), which leads to the generation of tensile stress in the sandstone sample near the coal–rock interface. When the tensile stress reached the tensile strength of the sandstone, a tensile crack initiated at the coal–rock interface where the tensile stress was a maximum and propagated upwards with axial loading. This is the mechanics of the tensile fracture that occurred in the rock sample of a coal–rock specimen that was subjected to uniaxial compression.

**Figure 6:**
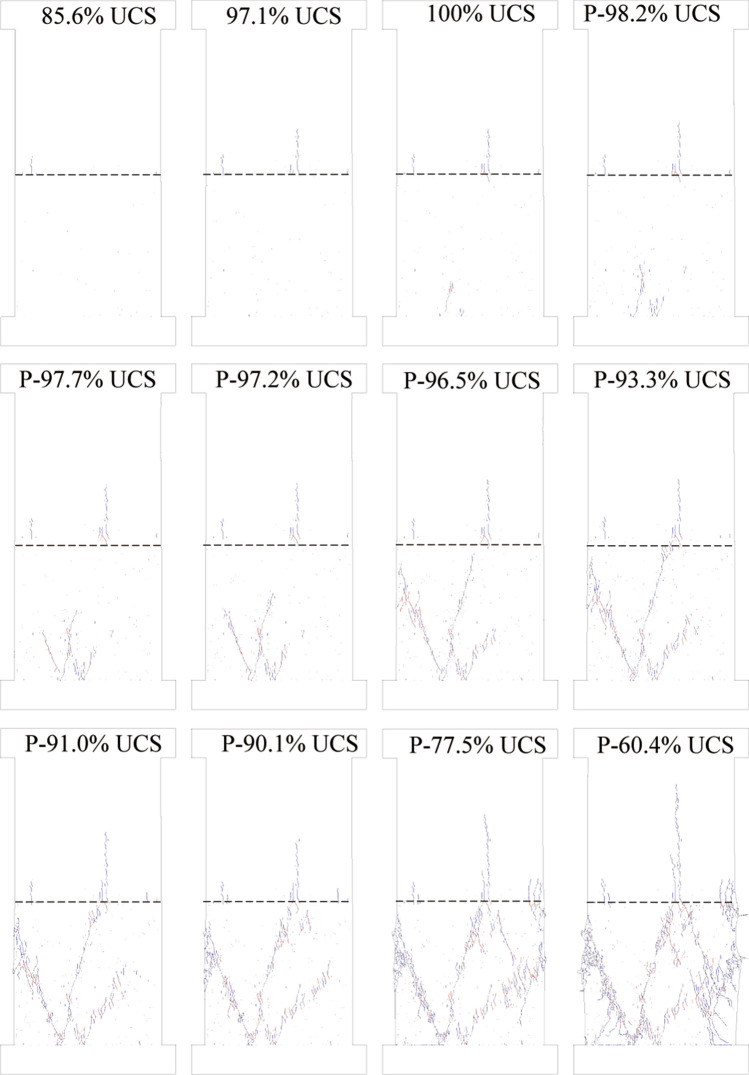
Simulated failure of coal–rock specimen subjected to unconfined compression. The number in front of UCS indicates the ratio of axial stress to the peak value. P indicates post-peak. Shear cracks showed in red and tensile cracks in blue. The black dotted line indicates the coal–rock interface.

**Figure 7:**
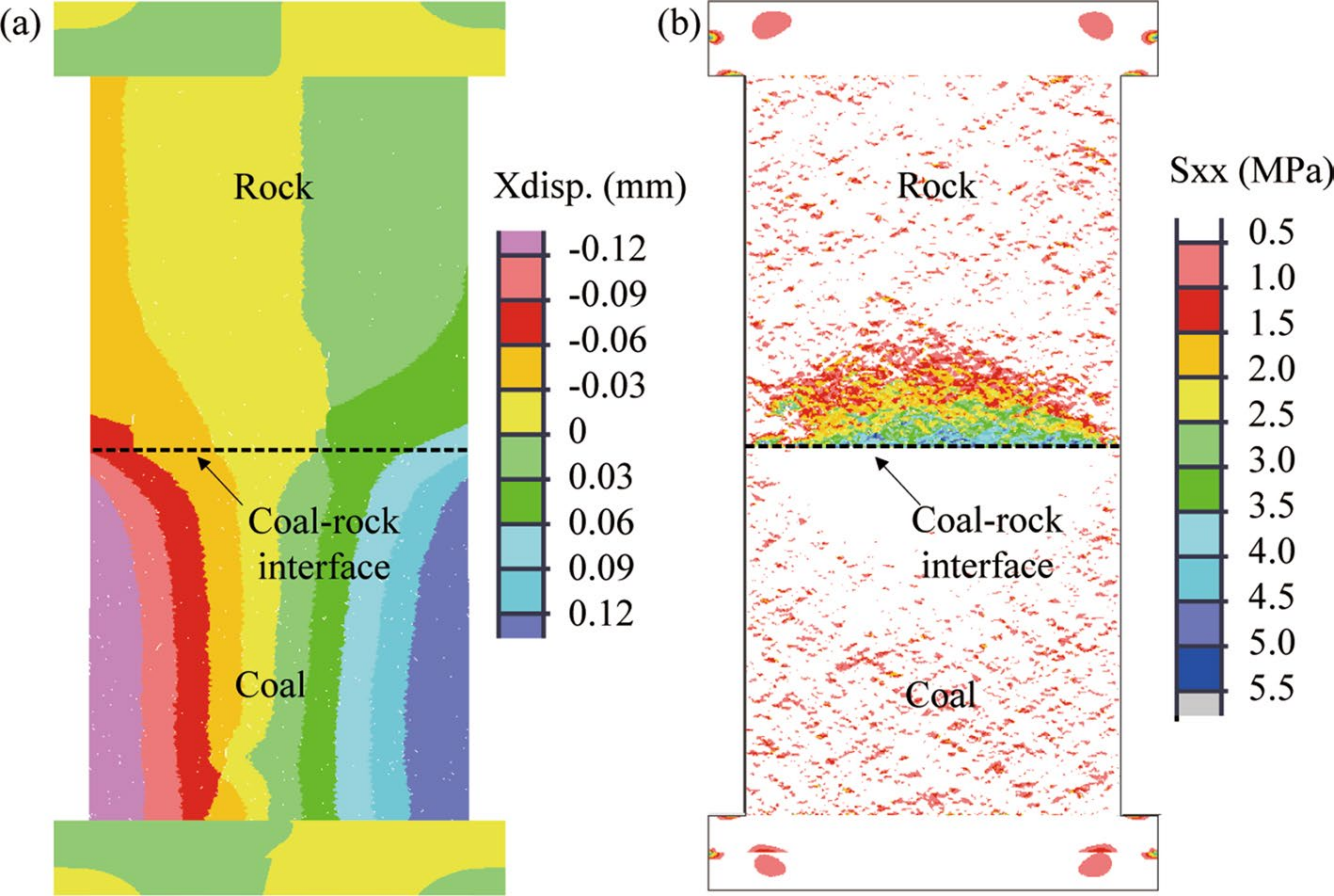
Simulated deformation and horizontal stress field before the generation of a tensile crack in the rock.

To demonstrate this postulate of sliding-induced tensile fracture, we created a numerical model to simulate the uniaxial compression test on a coal–rock specimen. Figure 6 shows the simulated failure of a coal–rock specimen that was subjected to uniaxial compression. As the axial load increased, the lateral strain on the coal side of the coal–rock interface increased more rapidly than that on the sandstone side (see Fig. 7a), which resulted in the generation of tensile stress within the sandstone (see Fig. 7b). The direction of the tensile stress was parallel to the coal–rock interface. When the tensile stress reached the tensile strength of the sandstone, cracks were initiated at the coal–rock interface within the sandstone at the loading of 85.6% UCS. As the loading continued and reached a peak, localized shear cracks occurred at the bottom of the coal and the coal–rock interface. Beyond the peak, shear cracks propagate significantly, which resulted in three shear bands. Two shear bands propagated to the lateral boundaries of the coal sample, and one cut through the coal and terminated at the coal–rock interface. The shear band terminated at the same location of the coal–rock interface where the tensile cracks initiated within the sandstone sample. This behavior is likely attributed to the fact that the generation of tensile cracks alters the local stress field at the periphery of the coal–rock interface. A comparison of Figs. 5 and 6 show that the numerical result agreed well with the laboratory result in terms of the initiation and propagation of tensile fractures within the sandstone sample, the intersection of the shear band in the coal and the tensile fracture in the sandstone, and the failure pattern of the coal–rock specimen.

**Figure 8:**
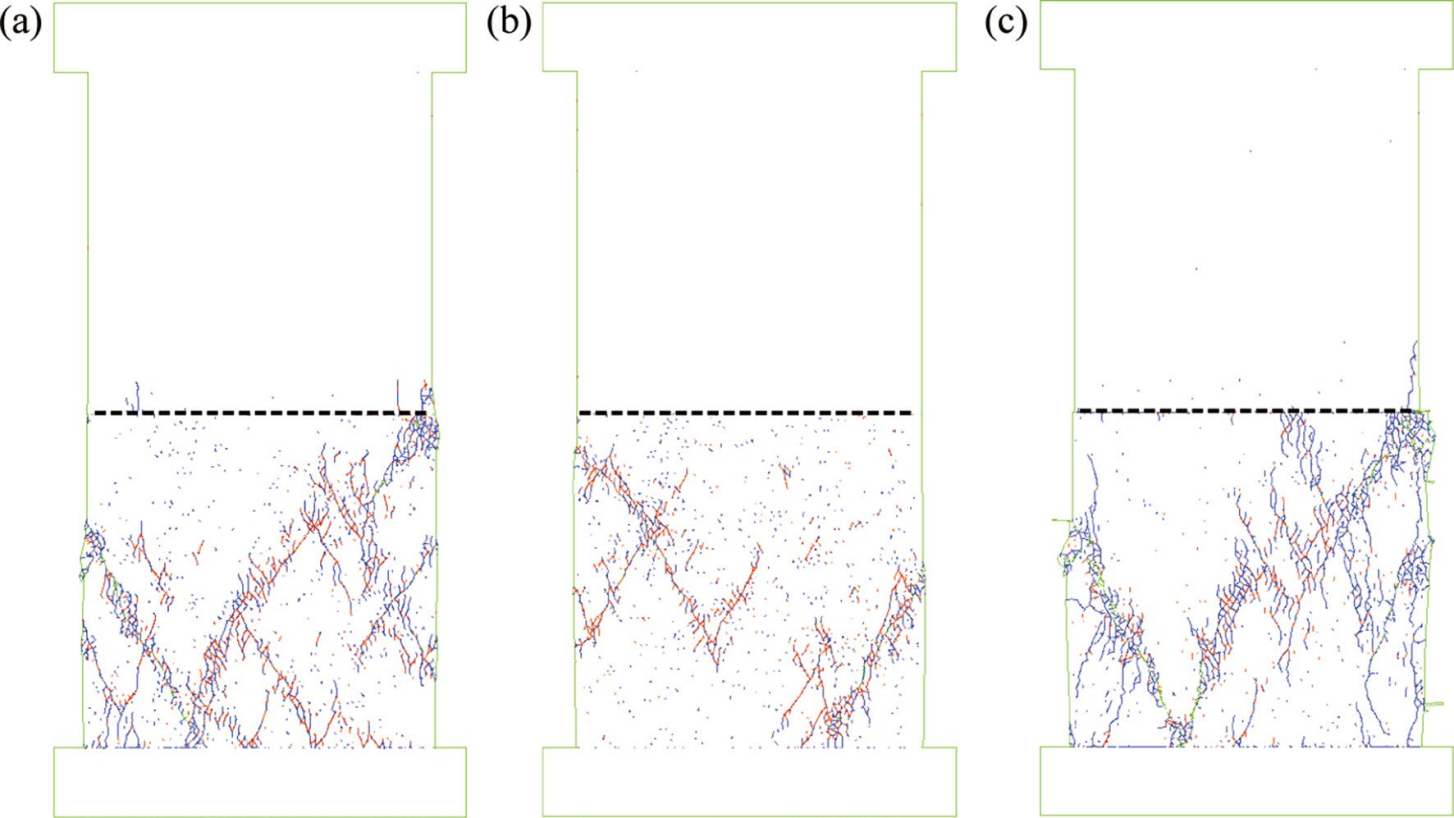
Effect of confining pressure and friction coefficient on coal–rock interface on failure pattern of coal–rock specimens. Tensile cracks in blue and shear cracks in red. Black dotted line indicates the coal–rock interface. (**a**) 2 MPa confining pressure, (**b**) 5 MPa confining pressure, (**c**) zero friction coefficient of coal–rock interface. The black dotted line indicates the coal–rock interface.

**Figure 9:**
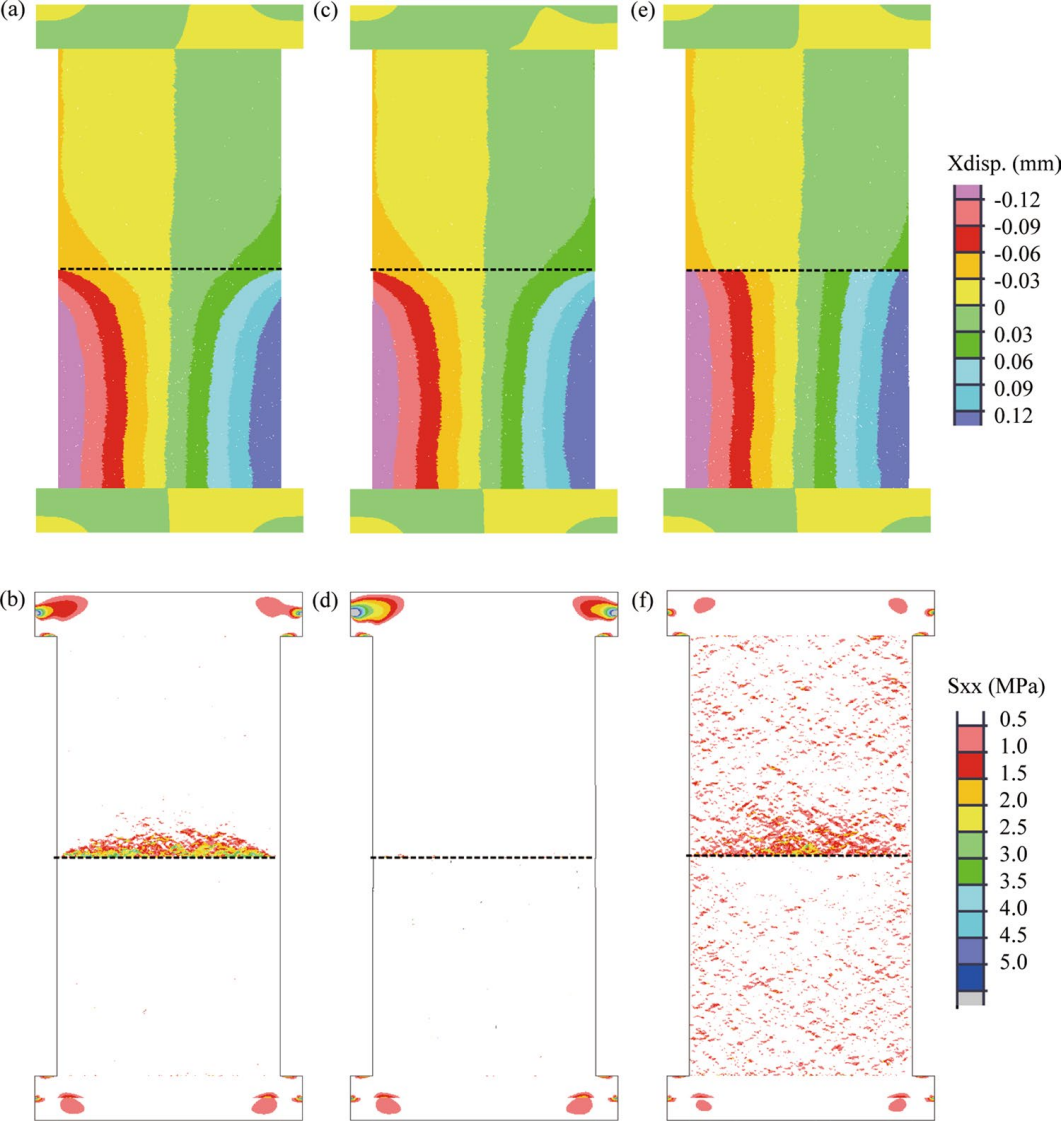
Effect of confining pressure and friction coefficient on deformation and horizontal stress field. (**a**) and (**b**) 2 MPa confining pressure, (**c**,**d**) 5 MPa confining pressure, (**e**,**f**) zero friction coefficient of coal–rock interface. The black dotted line indicates the coal–rock interface.

To show that the tensile fracture within the sandstone sample was caused by the tensile stress from the frictional drag along with the coal–rock interface, the numerical model was rerun with different confining pressures. As shown in Fig. 8a, under a low confinement of 2 MPa, tensile cracks still initiated at the coal–rock interface within the sandstone because of the uncoordinated deformation along with the coal–rock interface, see Fig. 9a. However, the propagation of tensile cracks was restrained by the confining pressure, see Fig. 9b. Under relatively high confinement of 5 MPa, the tensile stress from frictional drag (Fig. 9c) was offset significantly by the confining pressure (Fig. 9d), and no tensile cracks initiated at the coal–rock interface, see Fig. 8b. This finding was consistent with the laboratory results where no tensile fracture was observed within rock samples of coal–rock specimens under triaxial compression^39^.

It can be postulated that the tensile stress from frictional drag along the coal–rock interface can be eliminated if no friction exists on the interface. To demonstrate this hypothesis, we reran the numerical model with a zero-friction coefficient for the coal–rock interface. As shown in Fig. 8c, no tensile cracks were generated within the coal because the tensile stress along with the coal–rock interface was subtle (Fig. 9f), even though uncoordinated deformations occurred along with the coal–rock interface, see Fig. 9e.

### Unstable coal failure

**Figure 10:**
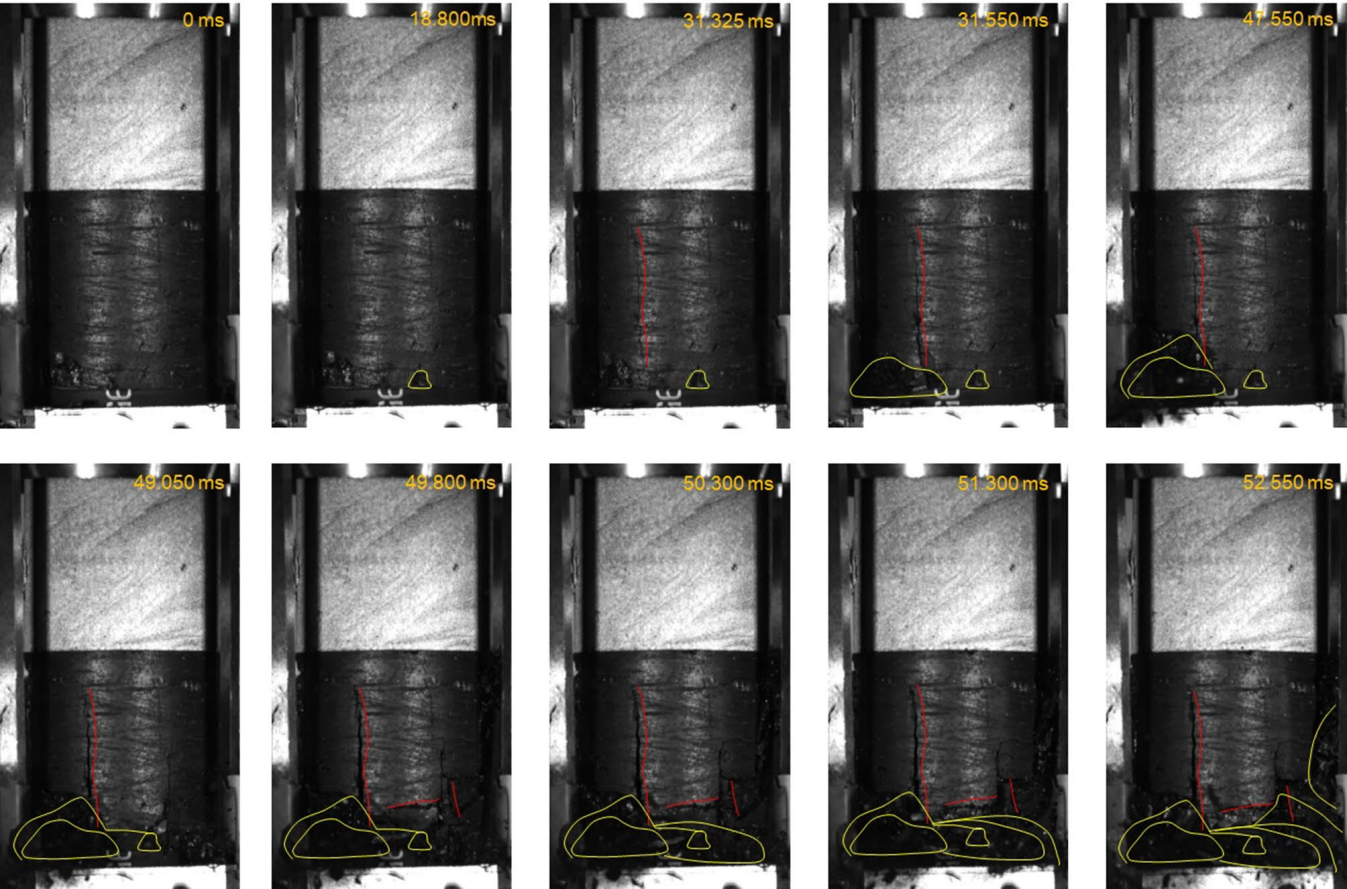
Snapshots showing unstable failure of coal sample in coal–rock specimen S-3 subjected to uniaxial compression. Yellow lines indicate the region of coal fragment ejection. Red lines indicate fractures.

A typical failure pattern of the coal–rock specimens under uniaxial compression was that the coal sample failed in an unstable way with coal fragment ejection, see Fig. 10. This pattern was observed in all three tested coal–rock specimens by using the high-speed camera, and was compared with the splitting or shear failure of pure coal specimens with a stable failure pattern, which is a typical failure pattern of brittle coal specimens under uniaxial compression^40–42^. This phenomenon can be explained by using LMS theory^24^. The conceptualization of mine stiffness has largely been framed by using analogies from post-peak unloading curves in laboratory tests and the comparable unloading of a mine pillar. If the host rock (loading system) relative to a mine pillar is too “soft”, the energy that is released from the surrounding rock during yielding of a pillar will exceed that at which the pillar can absorb and a burst will occur. The key concept of the LMS theory is that the energy that is released from the surrounding rock mass caused unstable mine pillar failure^25,43,44^. For the coal sample of the coal–rock specimens, the sandstone sample behaved like the surrounding rock. As the load increased, the strain energy accumulated gradually and was stored in the sandstone sample. When the load reached a peak value and the coal sample started to fail, the strain energy that was stored in the sandstone sample was released suddenly and transferred into the coal sample, which resulted in a violent failure.

**Figure 11:**
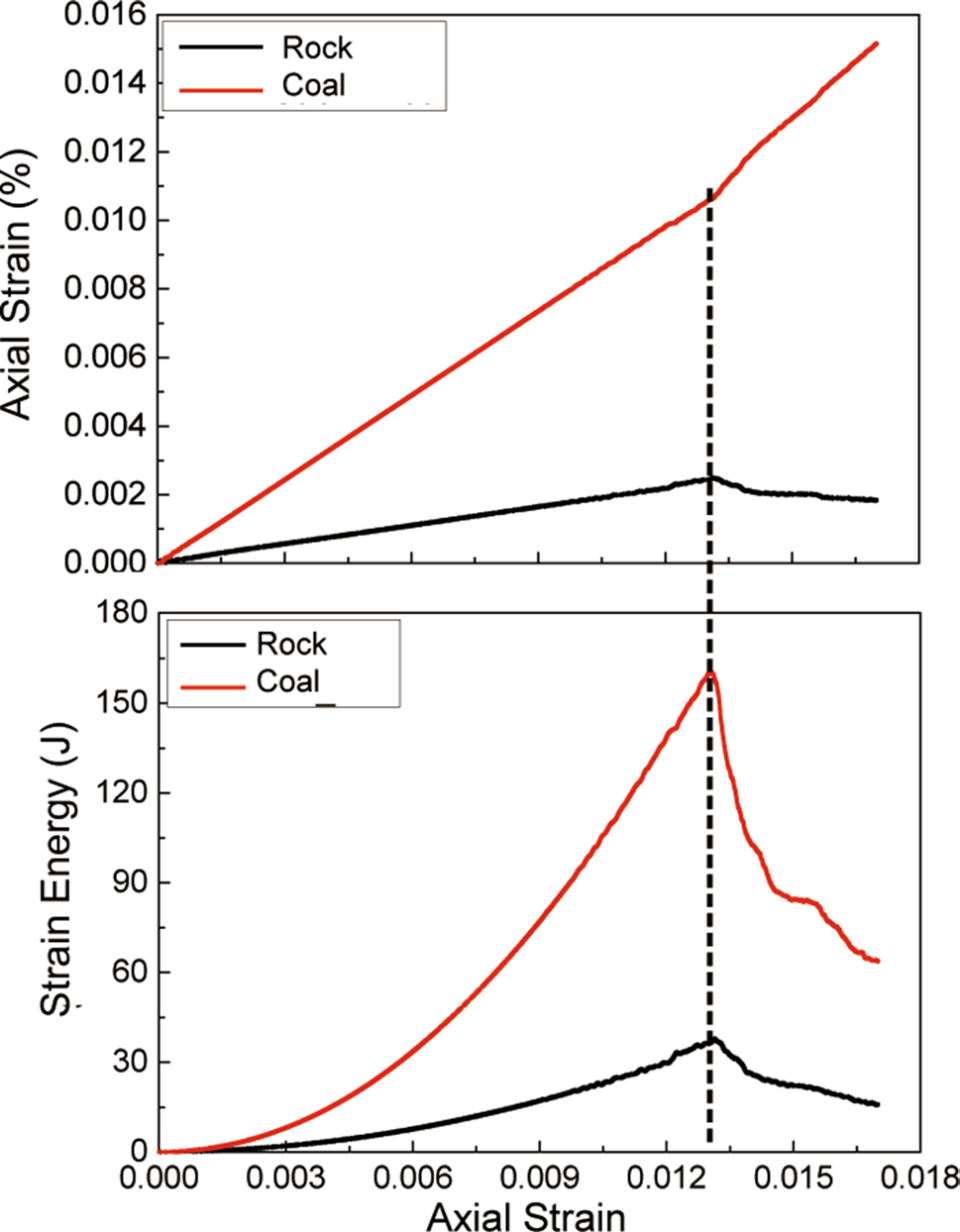
Changes in axial strain and strain energy with axial strain during unconfined compression tests on coal–rock specimens.

**Figure 12:**
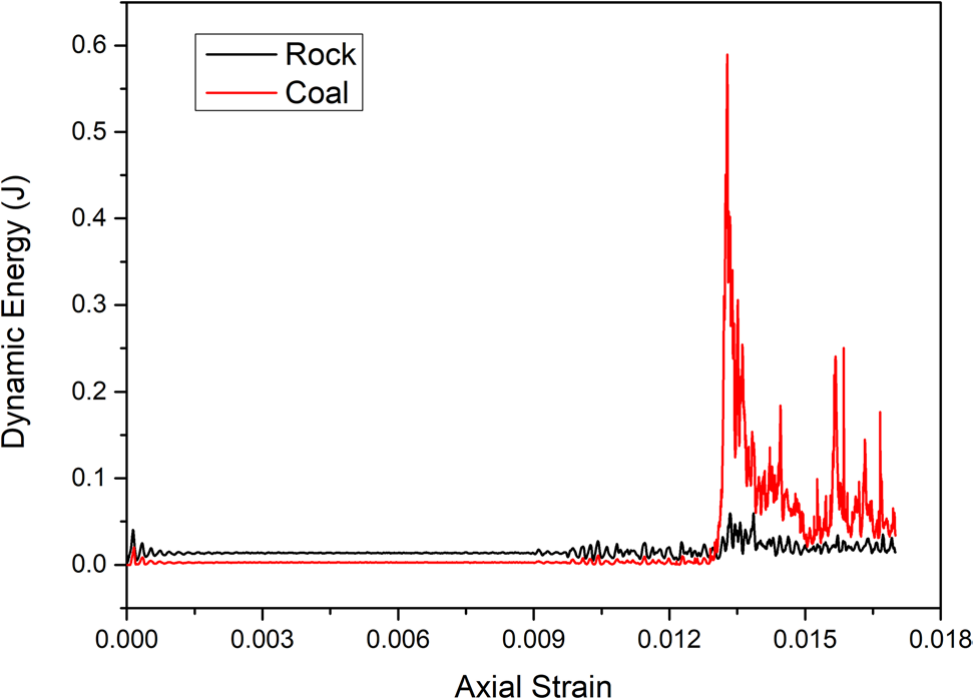
Changes in kinetic energy with axial strain during unconfined compression tests on coal–rock specimens.

We used the numerical model to evaluate the strain energy that was stored and released in the coal–rock specimen under uniaxial compression. Figure 11 illustrates how the strain and strain energy changed with loading. As the axial load increased, the axial strain in the coal and sandstone increased gradually. Because the elastic modulus of the coal was much lower than that of the sandstone, the axial strain and strain energy of the coal were significantly greater than that of the sandstone. After the peak stress was reached, the axial strain of the coal sample tended to increase because of the failure. In contrast, the axial strain of the sandstone decreased significantly, and the axial compression was restored to a certain extent, which indicates a significant elastic energy-releasing process. The strain-energy curve shows that the strain energy in the coal sample dropped sharply after reaching the peak stress, and indicates that part of the strain energy that was stored in the loading phase was consumed by shear and tensile failure, and part was converted into dynamic energy for coal fragment ejection, see Fig. 12. The strain energy in the sandstone was reduced significantly. A small part of the strain energy that was stored in the loading stage was consumed by tensile failure, and more were transferred to the coal sample, which exacerbated the unstable coal failure. This result explains why coal–rock specimens exhibit a higher burst tendency than pure coal specimens^45^.

## Conclusions

Under unconfined compression, the rock that is more competent than the coal has a significant impact on the deformability and a subtle impact on the strength of the coal–rock combined body. For all three tested coal–rock combined specimens, the rock failed with a splitting pattern from a single tensile fracture that developed sub-parallel with the loading direction. The failure process that was filmed by a high-speed camera contradicted the hypothesis that the tensile fracture was caused by energy released from the failure of the coal sample or the extension of a crack that developed within the coal. The tensile fracture was attributed to frictional drag along with the coal–rock interface under identical axial loading, which resulted in significant tensile stress that was applied on the rock side. The tensile crack propagated stably at ~ 0.05 times the Rayleigh wave speed *c*_*R*_. The coal failed with an unstable pattern, which was characterized by the ejection of small pieces from the coal specimen surface. This behavior is attributed to the release of strain energy that was stored in the rock specimen when the coal fails. This excessive strain energy was transformed into dynamic energy during coal failure.

## Methods

### Numerical model of coal–rock specimen

The model was created by using the UDEC Trigon method in which the rock and coal samples were represented as an assembly of triangular blocks that were bonded through contacts^46^. Each triangular block was elastic and was represented as a few finite-difference meshes. The failure of rock and coal was represented through tensile or shear cracking along the contacts between the blocks, depending on the stresses that were applied to the contacts. This UDEC Trigon method can simulate the initiation, coalescence and propagation of cracks in quasi-brittle geomaterials^47^. The properties of the blocks and contacts that were used to simulate the sandstone and coal were calibrated against Young’s modulus and the UCS of the sandstone and the coal, respectively. The calibration was achieved by performing a series of uniaxial compression tests with a UDEC Trigon model with a standard model size and identical mesh size with the coal–rock model. The calibrated properties are listed in Table 2.

**Table 2:** Mechanical parameters used in the coal-rock combined model.

Parameters	Coal	Sandstone	Rock–coal interface
Young’s modulus *E* (GPa)	2.2	11.5	–
Poisson’s ratio υ	0.28	0.26	–
Shear stiffness *K*_s_ (GPa/m)	1,500	7,504	4,000
Normal stiffness *K*_n_ (GPa/m)	3,750	18,760	10,000
Cohesion c (MPa)	8.2	15.2	0
Friction angle Ф (^o^)	30	34	30
Tensile strength σ_t_ (MPa)	2.2	4.2	0

### Energy calculation in the numerical model

For a given domain of material (e.g., the sandstone sample), the elastic strain energy $W_{e}$ can be calculated as

1 $$ W_{e} = \sum {E_{b} } + \sum {E_{c} } $$

where $\sum {E_{b} }$ is the sum of elastic energy stored in all the blocks in the domain, and $\sum {E_{c} }$ is the sum of elastic energy stored in all the contact in the domain.

For a given block, the elastic energy $E_{b}$ is determined by

2 $$ E_{b} = \frac{A}{2E}\left[ {\sigma_{1}^{2} + \sigma_{2}^{2} + \sigma_{3}^{2} - 2\upsilon \left( {\sigma_{1} \sigma_{2} + \sigma_{1} \sigma_{3} + \sigma_{2} \sigma_{3} } \right)} \right] $$

where $\sigma_{1}$, $\sigma_{2}$ and $\sigma_{3}$ are the three components of the principal stress, $A$ is the area of the block, $E$ and $\upsilon$ is the Young’s modulus and Poisson’s ratio of the block, respectively.

For a given contact, the stored elastic energy $E_{c}$ is composed of four components for elastic energy in shear ($U_{js}$), compression ($U_{jc}$), tension ($U_{jt}$), and energy dissipated in slip ($U_{jf}$).

3 $$ {\text{If}}\;f_{n} < 0,\;U_{jt} = - \frac{1}{2}\left( {f_{n} + f_{n}^{\prime } } \right)u_{n} $$

4 $$ {\text{If}}\;\;f_{n} \ge 0,\;\;U_{jc} = \frac{1}{2}\left( {f_{n} + f_{n}^{{\prime }} } \right)u_{n} $$

5 $$ {\text{If}}\;\;f_{s} < f_{s\max } ,\;\;U_{js} = - \frac{1}{2}\left( {f_{s} + f_{s}^{{\prime }} } \right)u_{s} $$

6 $$ {\text{If}}\;f_{s} \ge f_{s\max } ,\;\;U_{jf} = \frac{1}{2}\left( {f_{s} + f_{s}^{{\prime }} } \right)u_{s} $$

where $f_{n}$ and $f_{n}^{{\prime }}$ are the current and previous normal forces at the contact, respectively; $f_{s}$ and $f_{s}^{{\prime }}$ are the current and previous shear forces at the contact, respectively; $u_{n}$ and $u_{s}$ are the incremental normal and shear displacement at the contact over the current time step, respectively, and $f_{s\max }$ is the shear stress at which the contact meets the slip condition.

The kinetic energy $E_{k}$ in the rock and the coal was calculated by summing the kinetic energy of each block representing the rock and the coal:

7 $$ E_{k} = \sum\limits_{{}} \frac{1}{2} mv^{2} $$

where $m$ is the mass of the block and $v$ is the velocity of the blocks at the current time step. It should be noted that the calculated $E_{k}$ was implicit because the time was implicit in the static calculation model. But it is still meaningful when representing the evolution of the kinetic energy as loading.

## Data Availability

The data that support the findings of this study are available from the corresponding author upon reasonable request.
